# TBH1: 12 000-year-old human skeleton and projectile point shed light on demographics and mortality in Terminal Pleistocene Southeast Asia

**DOI:** 10.1098/rspb.2025.1819

**Published:** 2025-08-27

**Authors:** Christopher M. Stimpson, Alex Wilshaw, Benjamin Utting, Nguyen Thi Mai Huong, Nguyen Thi Hao, D.L. Vu, Tharsika Vimala, Hugh McColl, Emily M. Breslin, Eppie R. Jones, Ruairidh Macleod, Rachael Holmes, Shawn O'Donnell, Thorsten Kahlert, Sinh Pham Khanh, Bui Van Manh, Eske Willerslev, Ryan J. Rabett

**Affiliations:** ^1^ Oxford University Museum of Natural History, Oxford University, Oxford OX31 3PW, UK; ^2^ Bird Group, Natural History Museum, Tring, Hertfordshire HP23 6AP, UK; ^3^ School of Biological and Environmental Sciences, Liverpool John Moores University, Liverpool L3 3AF, UK; ^4^ Human Origins Program, National Museum of Natural History, Smithsonian Institution, P.O. Box 37012, Washington DC 20560, USA; ^5^ Institute of Archaeology, Vietnam Academy of Social Sciences, Hanoi, Hoan Kiem, Vietnam; ^6^ Tràng An Landscape Complex Management Board, Ninh Binh, Ninh Binh Province, Vietnam; ^7^ Lundbeck Foundation GeoGenetics Centre, University of Copenhagen, Copenhagen, Denmark; ^8^ Smurfit Institute of Genetics, Trinity College Dublin, Dublin, Ireland; ^9^ Department of Zoology, University of Cambridge, Cambridge, Cambridgeshire CB2 3EJ, UK; ^10^ Research Department of Genetics, University College London, London WC1E 6BT, UK; ^11^ School of Geography, Geology and the Environment, University of Leicester, Leicester LE1 7RH, UK; ^12^ Royal Botanic Gardens Kew, Richmond TW9 3AE, UK; ^13^ Archaeology & Palaeoecology, School of Natural & Built Environment, Queen's University Belfast, Belfast BT7 1NN, UK; ^14^ Department of Tourism, Ninh Binh, Ninh Binh Province, Vietnam; ^15^ Institute for Hellenic Culture & the Liberal Arts, The American College of Greece, Athens, Greece

**Keywords:** human osteology, mtDNA, conflict, anthropology, cultural evolution, trauma

## Abstract

The paucity of well-preserved and dated Pleistocene human remains impedes investigation of demographics and interactions in Late Pleistocene populations in Southeast Asia. Here, we report TBH1, an exceptionally well-preserved approximately 35-year-old male skeleton dated 12 500–12 000 years before present that provides rare insights into these debates. Superior preservation permitted detailed testing of different models of biological affinity and recovery of the earliest mitochondrial DNA evidence from Vietnam. Morphometric analyses indicated an affiliation with extant Southeast Asian Island populations, but with closest overall affiliation with regional Late Pleistocene data. Mitochondrial DNA sequencing showed unambiguous clustering within the M macrohaplogroup and a relationship with the early hunter–gatherer populations of South and Southeast Asia. While osteological analysis indicated good health during life, localized trauma to an accessory cervical rib was detected together with a small quartz flake with characteristics of a micropoint—an exotic technology within existing paradigms—in the immediate superio-posterior thoracic region. A case for a premortem timing for this injury, inflicted by the artefact, is presented. The trauma and subsequent infection are the likely cause of death and, to our knowledge, the earliest indication of interpersonal conflict from mainland Southeast Asia.

## 1. Introduction

The current paucity of well-preserved and reliably dated Pleistocene human skeletal and genetic remains, particularly crania [1–3], is a major impediment for detailed investigation of demographics, biological affinities and human dispersals in Late Pleistocene Southeast Asia [4–6]. While current evidence indicates that anatomically modern humans were present in Southeast Asia by approximately 70 ka (1000 years ago) [7,8], there are two ‘classical’ competing hypotheses on the ancestry of most modern East Asian populations. The first favours the persistence of indigenous (‘Hòabìnhian’) populations of hunter–gatherers who eventually developed agriculture independently of gene flow with any later, incoming populations. The second, the ‘two-layer hypothesis’, proposes instead that East Asian populations from the north migrated into Southeast Asia approximately 4 ka ago and introduced rice and millet [9,10]. Genetic investigations suggest that both contentions are oversimplifications [11].

Modelling potential scenarios of interaction between indigenous hunter–gatherers and incoming agricultural communities, for example, is also hampered by a lack of evidence and reliant on the appearance of cemetery sites, and is thus restricted to the Mid-Holocene, approximately 6 ka onwards [9,10,12,13]. Here, we present analyses of a remarkably well-preserved and near-complete human skeleton, TBH1, recovered from an anthropogenic midden deposit in a cave site in northern Vietnam, which provides rare insights into the nature of, and interactions within human populations in the Terminal Pleistocene, approximately 12 ka.

**Figure 1.**
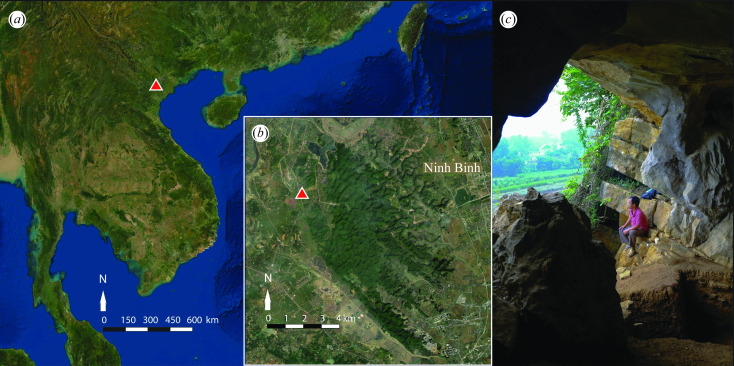
Tràng An and Thung Binh. (*a*) Location in Southeast Asia, (*b*) Thung Binh 1, the Tràng An karst massif and Ninh Binh and (*c*) looking south over trench 2 in the cave entrance. Map (ESRI Satellite base map, EPSG: 3857-WGS 84 Pseudo-Mercator projection) produced in QGIS [21]. Photograph: C. M. Stimpson.

**Figure 2.**
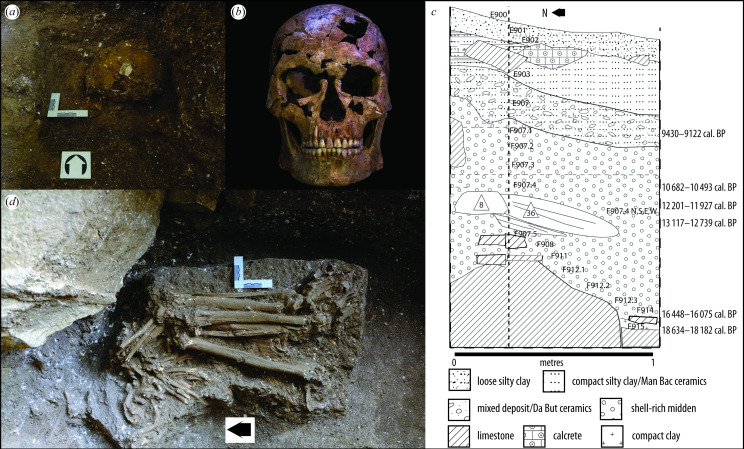
TBH1. (*a*) Collapsed skull in preparation for block-lifting, (*b*) skull after reconstruction, (*c*) representative west-facing section, showing calibrated radiocarbon dates (calibrated before present (cal. BP)) and stratigraphic position of TBH1 and, (*d*) exposure of post-crania prior to recovery. Arrows indicate north. Reconstruction: A. Wilshaw; photographs and drawing: C. M. Stimpson.

TBH1 was recovered between December 2017 and April 2018 from Thung Binh 1, a cave site in Tràng An Landscape Complex World Heritage Site, Ninh Binh Province, Vietnam. The environs and archaeological record of Tràng An have been described in detail elsewhere [6,14–20] but in brief, Thung Binh 1 is located within an isolated limestone hill on an alluvial plain to the northwest of the Tràng An massif (figure 1; see also [20]). The skull was found crushed (figure 2*a*) but in a condition that permitted its almost entire reconstruction, with complete dentition (figure 2*b*). The post‐cranial skeleton was also relatively well preserved (figure 2*d*); the pelvis and vertebrae were highly fragmented, but portions of most elements were recovered with fragmentation of the epiphyses of the post‐cranial bones.

The completeness of the skull permitted craniometric comparisons with local and regional datasets to test the competing models of biological affinity; these analyses were complemented by the analysis of mitochondrial DNA (mtDNA) evidence recovered from the petrosal bones. Skull completeness also permitted forensic facial reconstruction for exhibition purposes, using the ‘Combination Manchester Method’ [22,23] (electronic supplementary material, table S1, figure S1).

Examination of the cranial and post-cranial skeleton permitted inferences with regards to age, stature and health. Aside from a minor ankle injury, overall good health was indicated. As such, the occurrence of localized damage—in the form of fracturing—to an accessory cervical rib was therefore notable, as was recovery of a quartz micropoint (artefact no. 268) that is typologically unique in the site assemblage and, within existing paradigms, is exotic in terms of both time and place. This stone tool was found in direct association with elements from the superio-posterior thoracic region and this artefact and resultant trauma were likely significant contributing factors to mortality. The recovery of Late Pleistocene and Early Holocene human remains exhibiting trauma itself is a rarity [24,25]. To our knowledge TBH1 represents the oldest such case from mainland Southeast Asia.

## 2. Material and methods

### (a) Dating and recovery

**Figure 3.**
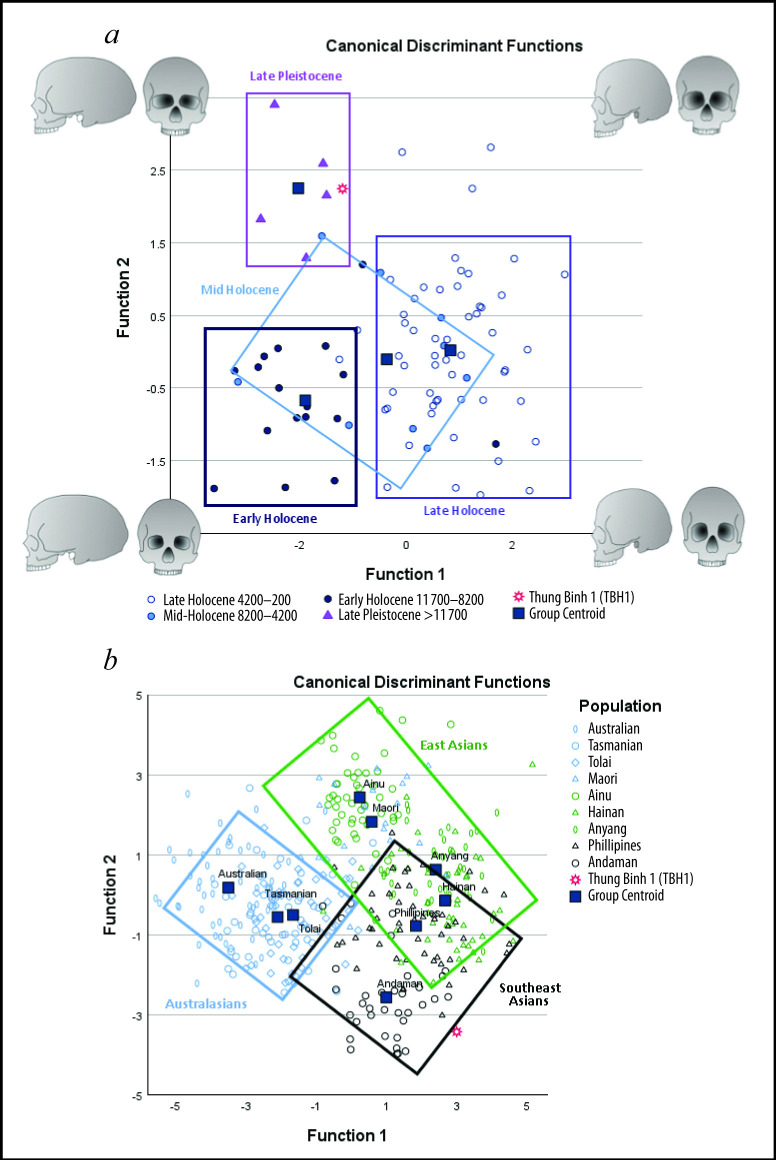
Discriminant Function Analysis (DFA). (*a*) Discriminant scores for the first two functions of the DFA for four prehistoric temporal groups: Late Pleistocene, Early Holocene, Mid-Holocene and Late Holocene and (*b*) discriminant scores for the first two functions of the DFA for extant geographical populations. Figure: A. Wilshaw.

Thung Binh 1 was excavated following natural stratigraphic boundaries, following an adapted single context recording system [26]. An area of approximately 5.5 m^2^ was excavated to an average depth of 1.60 m below the current cave floor, designated as trench 2 (electronic supplementary material, figures S2 and S3; see also [20]). The trench yielded a temporal sequence covering the twentieth century to 18 000 years before present (figure 2*c*). All radiocarbon dates were obtained from charcoal samples via accelerator mass spectrometry (AMS) at the AMS 14Chrono Centre Queen’s University Belfast and calibrated with calib 8.2 using the Intcal. 20 calibration curve [27]. Calibrated radiocarbon dates are shown as two sigma ranges as ‘cal. BP’ (‘calibrated years before present’). Reference to cultural materials and six radiocarbon dates derived from charcoal, three of which were found in direct association with TBH1 (figures 2*c* and 3), provide a chronology for the sequence. An attempt at dating residual bone powder (0.5 g) from sampling of the left petrous bone from the skull for ancient DNA (aDNA) failed owing to a lack of collagen.

The uppermost layers (E900–E902) were comprised of loose, silty clay hardened by trampling and calcium carbonate deposition, and contained a mixture of modern and historical material. An underlying, more compact silty clay layer (E903) contained a moderate frequency of animal bone fragments, occasional large marine shells and sherds of coarse ceramics. Isolated human phalanges and teeth were also found in direct association with larger sherds, as well as standardized perforated shell discs, attributed to the final Neolithic or Bronze Age Phùng Nguyên and the earlier Man Bac culture approximately 4.5 to 3 ka cal. BP [28,29].

Layer (E903) was underlain by a diffuse deposit (E907), which proved to be a remnant of a layer eroded by water action, mixed with the top of the underlying aceramic, shell-rich (predominantly *Cyclophorous* spp.), midden deposit (F907 to F9014), from where TBH1 was recovered. Small, eroded and rounded sherds attributed to Da But pottery and dating between 7000 and 3000 cal. BP (Nguyen 2005), were recovered in (E907), together with elements from the underlying midden (F907). We inferred that a charcoal sample that yielded a date of 9430−9122 cal. BP (UBA-36018) was disturbed from the top of the midden, giving a minimum age for midden accumulation. The midden deposit (F907) where TBH1 was interred was comprised of occasional stone tools, numerous fragments of animal bone and frequent charcoal fragments (electronic supplementary material, figure S2). Three charcoal samples were recovered in association with TBH1 and were dated (electronic supplementary material, figure S3). Charcoal recovered from the base of the midden, approximately 0.8 m maximum depth below the level of the burial, returned a date of 16 448−16 075 cal. BP (UBA-40555).

The top of the cranium of TBH1 was exposed in this deposit at an elevation of 27.27 m while the elevation recorded beneath the feet was 27.03 m. The skeleton thus dipped along its long-axis (north–south) approximately −15°. Due to sediment conditions, all remains were block-lifted and micro-excavated off-site. The bones of the skeleton had clearly been compressed, with many non-recent breaks evident. The individual appeared to have been placed in a foetal position, possibly tightly bound. The presence and position of the distal arm elements near to the base of the sediment block containing the skull suggests that both arms were probably bent and flexed up in the burial, with the hands together underneath the head, with the front of the face resting on them. Later micro-excavation of the skull from its sediment block revealed that it was broadly positioned on its right side, facing downwards into the ground.

No clear burial cut, change in the sediment character or content was identified during excavation, though a diffuse ash-rich deposit was noted to the western side of the excavation during exposure of the post-crania. After exposure *in situ*, the skull was found to be highly fragmented and flattened (figure 2*a*) and had been subject to an impact or sustained heavy weight. It was recovered underneath a large rock (electronic supplementary material, figure S3); in light of the size and likely weight of the boulder and what appeared to be a corresponding void in the cave ceiling above the burial site, roof fall was a parsimonious explanation for the damage. The post-crania were relatively well preserved (figure 2*d*; electronic supplementary material, figure S3). TBH1 was consolidated and reconstructed using museum grade paraloid B72 in pure acetone. After analysis, the bones were packed individually and stored at a secure location in a locked climate-controlled cabinet in the care of the Tràng An Management Board.

### (b) Craniometrics

The skeleton was described, and measurements of the skull were taken following [30,31] to maximize comparability (electronic supplementary material, table S2) to other published data and avoid known methodological discrepancies between variables used in the different approaches [10,32,33]. All analyses were based on two-dimensional craniometric measurements due to the absence of available and appropriate comparative three-dimensional data. Only measurements considered reliable within the limitations of the reconstruction of TBH1 were used. The data were prepared and analysed using IBM SPSS 29 [34]. Three comparative analyses were carried out using enter method multivariate DFA, with prior probabilities computed from group sizes and leave-one-out as a cross-validation to reduce potential overestimation of the DFA’s success [35]. TBH1 was entered as an ungrouped case, in order to give an indication of affinity. The data were tested for multivariate normality, homogeneity of variance–covariance, outliers and trait correlation to ensure that the underlying statistical assumptions of DFA were not violated, prior to the analyses being performed. The first DFA used 11 measurements common across a comparative sample made up of 87 published prehistoric crania from across Southeast Asia [10]. These were assigned to four temporal groups based on published radiometric dates or researcher-based age assessments based on the characteristics of sites: Late Pleistocene (>11.7 ka), Early Holocene (11.7−8.2 ka), Mid-Holocene (8.2−4.2 ka) and Late Holocene (4.2−0.2 ka). The measurements for this analysis, which represent both the neurocranium and viscerocranium, are based on the definitions of [36] and include: Maximum Cranial Length (M1), Maximum Cranial Breadth (M8), Minimum Frontal Breadth (M9), Basion-Bregma Height (M17), Upper-facial Height (M48), Orbital Breadth (M51), Orbital Height (M52), Nasal Breadth (M54), Nasal Height (M55), Frontal Chord (M43) and Frontal Subtense (M43c). One case (Gua Cha) was removed as an outlier which violated the underlying assumptions in respect of one variable (Nasion-Prosthion Height (NPH)), thus 86 cases were included in the final DFA.

Two further DFAs examined TBH1 in relation to recent populations taken from the Howell’s dataset [37]. Nine populations were chosen for their geographic proximity to TBH1 and reflect recent diversity of Southeast Asian (Andaman Islands, Philippines), East Asian (Ainu from Japan, Hainan and Anyang Chinese) and Australasian populations (Australian, Tasmanian, Tolai and Maori). All measurements conformed to the definitions of [30] and a total of 393 cases were included in the analyses. The first analysis used only 10 measurements, to reflect those used in the DFA of prehistoric crania, minus Martin’s [36] Minimum Frontal Breadth (9) which does not exist in Howell’s [37] dataset. The second analysis included all 26 reliable measurements commonly available from TBH1 and the Howell’s dataset.

### (c) mtDNA analysis

Authorized genetic sampling of both petrous bones was carried out by different laboratories. The left petrous was analysed (2018 to 2020) at the Smurfit Institute of Genetics, Trinity College, Dublin. The right petrous was analysed (2022 to date) at the Lundbeck Foundation GeoGenetics Centre, University of Copenhagen, focusing on the area of the otic capsule, which has been demonstrated to be the most likely part of the petrous to provide a high yield of endogenous DNA [38]. Next-generation double- and, subsequently, single-stranded libraries of the left petrous sample were undertaken on one and two lanes, respectively, of a HiSeq 2500 (Macrogen Inc., Korea) and mapped to the human reference genome assembly GRCh37 hg19. The single-stranded libraries were first analysed using the MiSeq System. In both cases, however, the low level of recovered endogenous DNA (the majority of sequences were found to be either bacterial or unidentifiable) meant that analysis yielded mixed signals, precluding reliable conclusions beyond the attribution of biological sex.

Analysis of the right petrous proved more productive and is on-going. For the mtDNA analysis, single-stranded next-generation libraries were prepared and sequenced on Illumina Novaseq 6000. Sequencing data were mapped to the human reference genome GRCh37 b37 first and then remapped to the revised Cambridge Reference Sequence (rCRS) to recover nuclear mitochondrial segments (NUMTs). Mapped reads were filtered for mapping quality 30 with SAMtools [39] and duplicates were removed with Picard MarkDuplicates (v.3.3.0). Variants were called using bcftools mpileup and call (v.1.18) [40] and filtered for sites covered by at least 5 reads with genotype quality of at least 25. The consensus sequences were produced using bcftools consensus (v.1.18). Haplogroups were determined with vgan haplocart (v.3.0.0) [41]. All sequences were aligned with mafft (v.7.525) [42] and sequences with less than 8000 missing sites were kept for analysis. The sequence alignment was constrained to the coding region within 577−16 023 bp (rCRS coordinates). The phylogenetic maximum likelihood analysis was carried out with raxML-NG (v.1.2.2) [43] under the model GTR+I+G4 with the accompanying options (--all –bs-trees 100).

### (d) Micropoint analysis

Metric attributes for artefact no. 268 were recorded with a set of Mitutoyo digital callipers and an electronic mass balance. Flake scars were identified and highlighted with the assistance of a hand lens.

## 3. Results

### (a) Dating

TBH1 was initially exposed approximately 0.9 m below the cave surface in a stratified context, designated (F907). Six calibrated radiocarbon dates derived from charcoal in the excavated sequence were in superposition and establish an intact chronology (figures 2*c* and 3). A sample from the overlying stratigraphic layer (E908) returned a date of 9430−9122 cal. BP (UBA-36018). Within (F907) a sample was recovered in sediment directly overlaying the elements of the leg (by 20 mm) and dated to 10 682−10 493 cal. BP (UBA-40556). In direct association with the body, a charcoal sample between the right humerus and the compacted skull returned a date of 12 201−11 927 cal. BP (UBA-36372) and a sample from the articulated assemblage of foot bones returned a date 13 117−12 739 cal. BP (UBA-38671). Charcoal from the base of the midden sequence, approximately 0.5 m below the burial, was dated to 16 448−16 075 cal. BP (UBA-386) and a sample from (F915), the first stratigraphic layer beneath the midden, returned a date of 18 634−18 182 cal. BP (UBA-40554). No clear burial cut or change in the sediment character potentially indicative of a cut was observed during excavation, although comparatively ash-rich deposits were recorded in association with and directly underlying TBH1. The absence of reversals within the spread of radiocarbon dates indicates against significant depositional mixing through burial activity.

### (b) Craniometrics

Approximately 75% of the skull could be reconstructed (figure 2*b*); the sphenoid, ethmoid, lachrymal, nasal conchae and vomer bones were either absent or too fragmentary/degraded for reconstruction. Areas of missing bone are distributed across the skull, but mainly in the posterior half of the right parietal and smaller areas on the anterior part of the left parietal and left superior frontal. The reconstructed viscerocranium is complete except for a fragment of the right medial part of the zygomatic and associated lateral supraorbital margin, as well as missing bone (approximately 10 × 10 mm) in the region of the supraorbital foramen. Parts of the alveolar clivus are degraded and the tooth roots are exposed.

In gross morphology, the cranial shape is ovoid. The neurocranium indicates a relatively long head, with a cranial index of 75. The mandible and dentition are complete except for the lateral half of the condyle articulation and a small fragment posterior to the left M3. The mandible is generally squared and broad both anteriorly and posteriorly; relatively high but short and overall, relatively robust. All teeth are present and fully erupted, including both maxillary and mandibular third molars. While the dimensions of the teeth are large compared with modern-day samples (electronic supplementary material, table S3), they are not significantly so for the period [1]. The teeth exhibit minor evidence of linear enamel hypoplasia and relatively heavy but unusual wear patterns to the lingual surface of both I^1^s, which is suggestive of extramasticatory forces acting on these teeth, possibly from plant processing.

The skull indicates a relatively large, long head with a broad frontal and wide, short orbits. In comparisons of 11 variables with prehistoric datasets, three Discriminant Functions were calculated, of which two showed significant differences between four temporal groupings (*χ*^2^_33_ = 104.476, *p* < 0.001; *χ*^2^_20_ = 33.970, *p* < 0.05; figure 3*a*). Correlations between the predictor variable and first function relied upon a negative correlation with cranial length and positive correlations with orbital and facial height, suggesting that the neurocranium becomes shorter through the Holocene and the upper face and orbits more elongated. The second function relied upon positive correlations with orbital and frontal breadth and the frontal chord, with the Late Pleistocene group having wide frontals and orbits that become narrower in all the Holocene groups. The discriminant scores for the first and second functions show that the Late Pleistocene, Early Holocene and Late Holocene groups exhibit relatively distinct morphologies and distinct areas of morphospace (figure 3*a*). The Mid-Holocene group is less distinct, and individuals generally conform either to the Early or Late morphotype as part of a Holocene trend towards a relatively wider neurocranium. Overall, DFA successfully predicted the outcome for 88.4% of grouped cases, with a cross-validation of 77.9%. TBH1 was consistently classified with the Late Pleistocene group (figure 3*a*).

In comparisons with extant datasets, eight discriminant functions were calculated, all of which showed statistical significance; only the first two results are reported here (*χ*^2^_208_ = 1878.686, *p* < 0.001; *χ*^2^_175_ = 1235.646, *p* < 0.001). Correlations between the predictor variable and first function relied upon a negative correlation with inferior malar length (IML) and accounted for 49.3% of variation between the groups. The second function relied upon positive correlations with cranial length (GOL) and orbital and biasterionic breadth (OBB, ASB) and accounted for a further 18.5% of variation between the groups. The discriminant scores for the first and second functions show that there is considerable overlap in morphology between the groups (figure 3*b*). Despite this, both the Southeast Asian and Australasian groups show distinctive variation. Overall, the DFA successfully predicted the outcome for 81.9% of grouped cases, with a cross-validation of 74.5%. Thung Binh 1 was consistently associated with Southeast Asian populations, and specifically with historical data from the Philippines. It should be noted, however, that this is the closest match given the variation incorporated within the model. If TBH1 is entered as an independent group, the morphospace is expanded extensively; TBH1 is more distant from any of the extant populations, than they are from each other. The difference in TBH1’s population affinity between this and the first DFA are likely due to the expanded set of variables (*n* = 26 and *n* = 10, respectively). It appears that specific size and shape of facial features, particularly the zygomatic bone, is important for differentiating the populations and this variation was not captured by the 10 variables used in the previous analysis.

### (c) mtDNA

**Figure 4.**
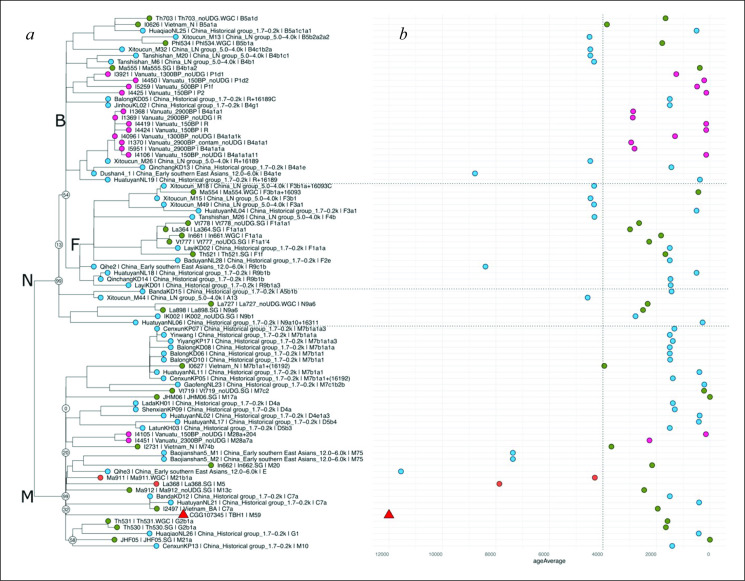
Phylogenetic maximum likelihood analysis of TBH1. (*a*) Maximum likelihood tree showing the phylogenetic clustering of TBH1 within haplogroup M. Colours indicate ancestry groups of each individual included in the analysis. TBH1 is indicated with a red triangle. Bootstrap values for the deepest splits are shown in white circles and (*b*) timeline displaying the age individuals along the *x*-axis. The timing of the farmer expansions approximately 4 ka has been annotated with a dotted vertical line. Figure: T. Vimala.

The sequencing of samples taken from the left petrous confirmed the presence of endogenous DNA (0.34%) but the low proportion and complexity of human reads and high levels of fragmentation (length distribution peaking in the range 30−40 base pairs) prevented further confident assessment beyond confirmation of sex as male. The mtDNA extracted from the right petrous (electronic supplementary material, figures S3 and S4) demonstrates that this individual carried the M59 mitochondrial lineage, the youngest of four basal haplogroups with a likely coalescence age to the most recent common ancestor of 27 594 ± 10 430 years ago [44]. This haplogroup falls within the same broad cluster as data from Gua Cha (Malaysia) and Pha Faen (Laos) [5] indicating that TBH1’s maternal lineage is affiliated with indigenous hunter–gatherer populations. Individuals associated with the later Austroasiatic and Austronesian migrations tend to carry F- and B-related haplogroups. The genetic placement within the M haplogroup cluster was further confirmed by a maximum likelihood-based phylogenetic analysis in the context of previously published Southeast Asian individuals [11,45–47] (figure 4).

### (d) Post-crania, pathology and quartz micropoint

**Figure 5.**
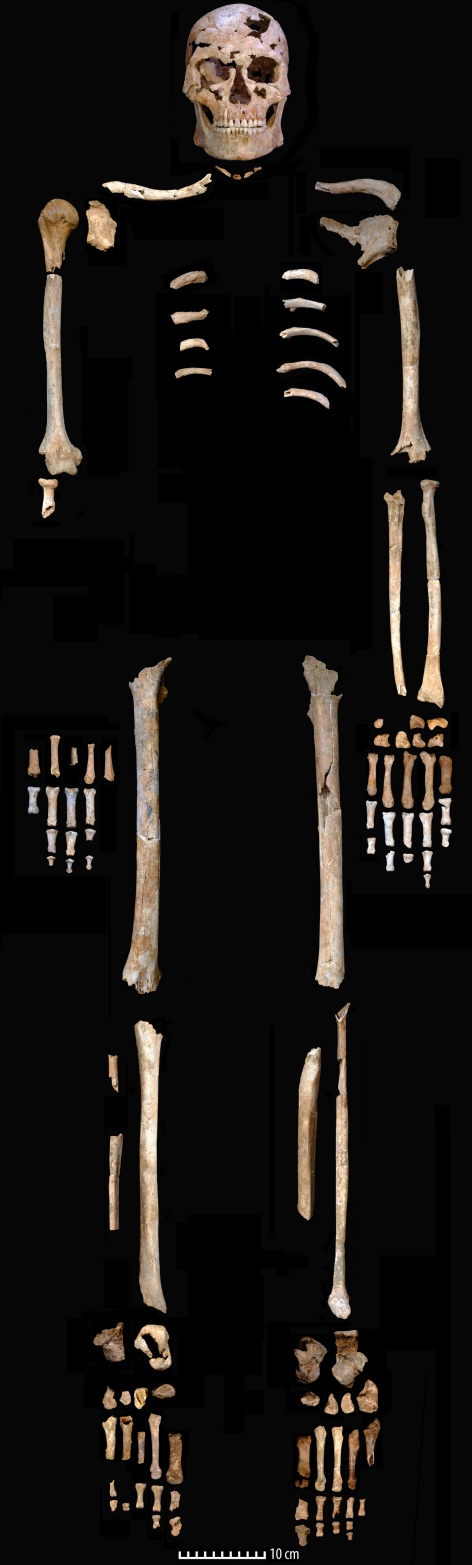
Elements of TBH1 in anatomical position. Scale: 10 cm. Photographs: C. M. Stimpson; reconstruction: A. Wilshaw.

The post‐cranial skeleton was relatively well preserved (figure 2*d*). The pelvis and vertebrae were highly fragmented, but portions of most elements were recovered (figure 5), with fragmentation of the epiphyses of the post‐cranial bones. There was also evidence of animal gnawing to the feet, yet the bones were not dispersed suggesting that the body was kept above ground for a time prior to burial; animals had access to the extremities but did not have opportunity to remove parts of the body.

TBH1 was also assessed as male, based on general robusticity and cranial attributes. Maxillary and mandibular third molars are fully erupted, indicating an adult over the age of 21 years. The observable portion of the medial epiphysis of the clavicle appears completely fused although only recently so at time of death; this, however, would indicate an individual over the age of 30 years. A small fragment of the iliac crest (approximately 50 mm) was preserved and is fully fused, indicating an age of over 22 years [48]. Two differing suture closure methods indicate a mean age at death of 34.7 years and 36.2 years (electronic supplementary material, table S5).

**Figure 6.**
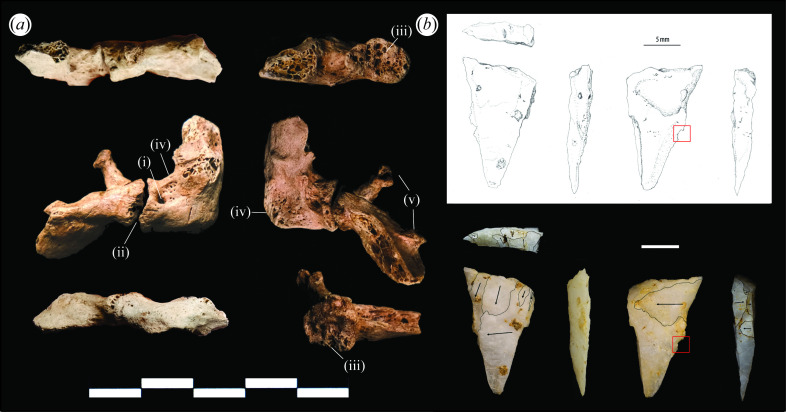
Fracture trauma, remodelling and evidence of infection to the accessory cervical rib from TBH1, and artefact no. 268, a quartz micropoint. (*a*) Accessory cervical rib, annotations: i—draining cloaca, ii—fracture site, iii—macro-porosity on false joint surface, iv— osteomyelitis and thickening of bone, v—tubercle and head of the rib with pseudo-articular surfaces (scale is 50 mm) and (*b*) artefact no. 268 with micro-notch (red square) and flake scar annotations (scale: 5 mm). Photographs and illustration: C. M. Stimpson; reconstructions: A. Wilshaw; flake scar annotations: B. Utting.

There is no specific equation to deal with Pleistocene human stature estimations, therefore equations based on extant Southeast Asian datasets were used in the calculation of stature. As a broad estimation, TBH1 would have been approximately 1.7 m tall (electronic supplementary material, table S6). A lack of marked pathologies indicates that TBH1 was relatively healthy throughout his lifespan. Most of the skeleton exhibited no evidence of trauma, with two exceptions. Firstly, light remodelling of the right calcaneous around the sustentaculum tali likely reflects a minor trauma (an impact, sprain or possibly minor arthritis) or ligament damage to the right ankle. Secondly, and most notably, a complete fracture with remodelling on a cervical rib (figure 6*a*).

Cervical (or supernumerary) ribs arise through congenital diversity. They are a variant present in a small proportion of the population (typically 0.2 and 1.0%); four subtypes have been identified [49–52] (electronic supplementary material, tables S4 and S7). The TBH1 cervical rib is incomplete (figure 6*a*). Siding is provisional owing to inherent variability of form in accessory ribs [52]. The pieces were, however, excavated from a sediment block containing right-sided elements and thus it is also likely to be from the right side. Based on element size and the apparent absence of interface with the first rib, we assign the element to Grüber subtype 2. Infection is suggested by the presence of a draining cloaca and further indicated by what appears to be the development of a false joint (pseudoarthrosis) between the fractured pieces of the rib (figure 6*a*). This is possibly due to soft-tissue intrusion into the fracture space preventing the union of the bone and indicates that TBH1 lived for several months after the injury occurred. The nature of the bone infection also points to an injury that caused an open fracture. Without effective treatment, this is likely to have led to bacterial and other forms of infection [53].

Artefact no. 268, a small triangular retouched quartz flake that some would describe as a micropoint (figure 6*b*; dimensions: 18.28 mm long, 8.92 mm wide, 3.04 mm thick and weight: 0.4 g), was identified in the project’s field laboratory during micro-excavation of a sediment block from context (F907.4) with both fragments of the cervical rib, fragments of the right scapula, a partial shaft of the right second rib and two fragments of spinous process from cervical vertebrae (C6 and/or C7). The artefact is made on opaque milky quartz. Of the 615 lithics recovered and analysed from the site, a total of 25 were manufactured on quartz. However, unique technological characteristics distinguish artefact no. 268 from the site assemblage and other local assemblages of comparable antiquity [54,55]. The absence of similar technology from within the Tràng An massif suggests it represents a non-local technological element. The piece exhibits a flat profile, imposed form and limited backing retouch, particularly along but not confined to the dorsal edge, matching some microlith criteria [56]. The steep dorsal retouch, size and form of the piece bear qualities of a triangular geometric microlith, though with a lesser degree of backing retouch. While a micro-notch to the longest edge (figure 6*b*—red square) cannot, in isolation, be taken as evidence of use as a projectile [57], micro-notching is considered to be diagnostic of impact damage and may indicate use as a barb [58,59]. Based on this and the form of the piece it is reasonable to suggest that it was hafted as part of a composite projectile system and of non-local origin.

## 4. Discussion

Craniometric analysis of TBH1 shows clear clustering with regional data for the Late Pleistocene, and extant Southeast Asian Island populations. Mitochondrial DNA analysis shows unambiguous clustering with macrohaplogroup M and early human colonizers of South and Southeast Asia [60,61]. Our findings contrast with recently published mtDNA evidence of two individuals (CCNM55 and CCNM24) from Con Co Ngua, which prior to this paper, represented the oldest mitogenomes from hunter–gatherer populations from Vietnam. Both of those individuals were assigned to haplogroup R0 affiliated with the N macrohaplogroup with closer maternal relatedness to Neolithic populations, than to early hunter–gatherers [62]. An influx of mitochondrial haplogroups with admixture between indigenous and incoming agricultural populations is not indicated until approximately 6.4 ka cal. BP [63,64]. Dated charcoal samples overlaying TBH1, in the same context and from the overlying stratigraphic layer, indicate that the skeleton predates 9.1−10.6 cal. BP; samples recovered in direct association with the skeleton returned dates 11.9−13.1 ka cal. BP. Thus, phenotypic and genetic data, together with radiocarbon dating are consistent in indicating that TBH1 derives from a Late Pleistocene indigenous hunter–gatherer population, with no indication of the influence of East Asian farmer populations.

Osteological analysis of the TBH1 skeleton revealed no significant pathologies or trauma, with the notable exception of a fractured and infected cervical rib. Recovery of a quartz micropoint in direct association with the right neck and shoulder elements supports its candidacy as the cause of this injury. While archaeological assemblages containing miniaturized projectiles have been reported from a variety of periods and geographic contexts [65,66], this technology has not yet been widely identified in Mainland Southeast Asia. Current known occurrences derive from island contexts and include an obsidian-dominated assemblage from Tron Bon Lei, Alor Island, approximately 12 ka cal. BP [67]; Liang Jon, East Kalimantan, dated to slightly older than 10.6−10.3 ka cal. BP [68]; and an industry-defining position within Toalean assemblages from later, Mid-Holocene contexts, e.g. approximately 4.8−4.2 ka cal. BP at the Leang Bulu Bettue rockshelter, Sulawesi [68–70]. A trend towards technological miniaturization in Southeast Asian assemblages is long recognized [71] and it is possible that simple and amorphous forms, which did not fit easily into traditionally prevailing lithic typologies, have drawn limited attention. Microlith-like components may have been subsumed within what are instead characterized as ‘small flake’ industries, such as have been recorded from sites in the Lingnan region of southern China [72] and in northern Vietnam, notably from the Nguom Rockshelter, approximately 100 km north of Hanoi. That site yielded a high proportion of small flake tools 20−30 mm long with marginal retouch [73,74]. Recent optically stimulated luminescence (OSL) dating suggests that the main assemblage at Nguom (Layers 7−8) was deposited between 18.5 ± 0.8 ka and 10.7 ± 0.6 ka (Layer 8, NG17-OSL 1) [75] and thus broadly similar in age to TBH1.

The trauma to the cervical rib is an exceptional find for the region specifically and this time period, more generally. Given the anatomical location of cervical ribs and the level of musculature around (though not attached to) them in adults, fracturing is most likely caused by direct, typically high-velocity, trauma (electronic supplementary material, text S4 ). On the basis of the evidence, a narrow penetrative impact is the only feasible cause of fracturing; anything larger than a small projectile would almost certainly have resulted in severe damage to the neck and likely to have been immediately fatal (e.g. including severance of the phrenic nerve). The evidence of infection on the cervical rib is consistent with septic arthritis [76,77] and demonstrates that TBH1 survived the initial injury, perhaps by a few months.

The typological nature of the micropoint, its uniqueness and damage profile offer a case for interpersonal violence. A growing literature covers the occurrence of interpersonal confrontation among prehistoric hunter–gatherer societies [78–81]; our study extends this record further back in to at least the final millennia of the Late Pleistocene.

## Data Availability

Datasets supporting this article have been uploaded as electronic supplementary material [82].
